# Complete Genome Sequences for 35 Biothreat Assay-Relevant *Bacillus* Species

**DOI:** 10.1128/genomeA.00151-15

**Published:** 2015-04-30

**Authors:** Shannon L. Johnson, Hajnalka E. Daligault, Karen W. Davenport, James Jaissle, Kenneth G. Frey, Jason T. Ladner, Stacey M. Broomall, Kimberly A. Bishop-Lilly, David C. Bruce, Henry S. Gibbons, Susan R. Coyne, Chien-Chi Lo, Linda Meincke, A. Christine Munk, Galina I. Koroleva, C. Nicole Rosenzweig, Gustavo F. Palacios, Cassie L. Redden, Timothy D. Minogue, Patrick S. Chain

**Affiliations:** a Los Alamos National Laboratory (LANL), Los Alamos, New Mexico, USA; b United States Army Medical Research Institute of Infectious Diseases (USAMRIID) Diagnostic Systems Division (DSD), Fort Detrick, Maryland, USA; c Naval Medical Research Center (NMRC)–Frederick, Fort Detrick, Maryland, USA; d Henry M. Jackson Foundation, Bethesda, Maryland, USA; e United States Army Medical Research Institute of Infectious Diseases (USAMRIID), Center for Genome Sciences (CGS), Fort Detrick, Maryland, USA; f United States Army Edgewood Chemical Biological Center (ECBC), Aberdeen Proving Ground, Aberdeen, Maryland, USA

## Abstract

In 2011, the Association of Analytical Communities (AOAC) International released a list of *Bacillus* strains relevant to biothreat molecular detection assays. We present the complete and annotated genome assemblies for the 15 strains listed on the inclusivity panel, as well as the 20 strains listed on the exclusivity panel.

## GENOME ANNOUNCEMENT

For several years the idea that biothreat and contamination detection methods need to be better characterized has been discussed (1–3). This led the Association of Analytical Communities (AOAC) International to compose a bacterial strain list for evaluation when designing molecular detection assays. This list, termed the Stakeholder Panel on Agent Detection Assays (SPADA), includes 15 inclusivity and 20 exclusivity *Bacillus* strains (4). As testing pertaining to these strains involves nucleic acid analyses, complete genome assemblies can further improve assays confidence, a major issue in both positive and negative results (5). Here, we describe complete genomes for all 35 strains.

Each microbial isolate genome was assembled using at least two data sets (specific data types and coverages are listed in the NCBI records): Illumina (short- and/or long-insert paired data), Roche 454 (long-insert paired data), and PacBio long reads. Short- and long-insert paired data were assembled in both Newbler and Velvet and computationally shredded into 1.5-kbp overlapping shreds. If PacBio coverage was 100× or greater, the data were assembled using PacBio’s Hierarchical Genome Assembly Process (HGAP) (6), all data were additionally assembled together in Allpaths (7). Consensus sequences from HGAP and Allpaths were computationally shredded into 10-kbp overlapping pieces. All shreds were integrated using Phrap. Possible misassemblies were corrected and repeat regions verified using in-house scripts and Consed for manual editing (8–10). All but one of the genomes were assembled into finished-quality complete genomes (11). Each genome assembly was annotated using an Ergatis-based (12) workflow with minor manual curation.

Genome assemblies range from 3.4 to 6.7 Mb (Table 1; smallest *B. coagulans* DSM 1 and largest *B. thuringiensis* subsp. *Morrisoni* HD 600) with up to 14 plasmids (mean, 2.9 ± 0.5) and G+C contents of 33 to 47% (only *B. coagulans* DSM 1 has a G+C content greater than 40%).

**TABLE 1:** *Bacillus* genomes^a^

Strain	Accession no.	Panel	AOAC no.	Assembly (bp)	No. of plasmids	G+C content (%)
*B. anthracis*						
Turkey32	CP009314–CP009316	I	BA15	5,505,298	2	35
2002013094	CP009900–CP009902	I	BA12	5,601,083	2	35
Ames_BA1004	CP009979–CP009981	I	BA5	5,503,969	2	35
BA1015	CP009542–CP009544	I	BA4	5,491,163	2	35
BA1035	CP009698–CP009700	I	BA10	5,487,253	2	35
Canadian bison	CP010320–CP010322	I	BA1	5,505,775	2	35
K3	CP009329–CP009331	I	BA6	5,504,993	2	35
Ohio ACB	CP009339–CP009341	I	BA7	5,498,337	2	35
PAK-1	CP009324–CP009325	I	BA3	5,403,381	1	35
Pasteur	CP009475–CP009476	I	BA13	5,294,803	1	35
RA3	CP009695–CP009697	I	BA11	5,489,869	2	35
SK-102	CP009462–CP009464	I	BA8	5,505,681	2	35
Sterne	CP009540–CP009541	I	BA14	5,409,120	1	35
V770-NP-1R	CP009597–CP009598	I	BA2	5,410,397	1	35
Vollum 1B	CP009326–CP009328	I	BA9	5,506,626	2	35
						
*B. cereus*						
03BB102	CP009317–CP009318	E	BANN13	5,448,107	1	35
D17	CP009299–CP009300	E	BANN7	5,590,358	1	35
03BB108	CP009634–CP009641	E	BANN14	6,450,959	7	33
3A	CP009593–CP009596	E	BANN2	5,642,300	3	35
ATCC 4342	CP009627–CP009628	E	BANN10	5,306,298	1	35
E33L	CP009965–CP009970	E	BANN6	5,846,781	5	35
FM1	CP009368–CP009369	E	BANN11	5,697,763	1	35
G9241	CP009589–CP009592	E	BANN12	5,720,073	3	35
S2-8	CP009604–CP009606	E	BANN1	5,642,468	3	35
						
*B. coagulans*						
ATCC 7050	CP009709	E	BANN18	3,366,995	0	47
						
*B. megaterium*						
ATCC 14581	CP009915–CP009921	E	BANN20	5,746,640	6	38
						
*B. mycoides*						
ATCC 6462	CP009689–CP009692	E	BANN19	5,637,053	3	35
						
*B. thuringiensis*						
Al. Hakam	CP009645–CP009651	E	BANN9	5,676,963	6	36
97-27	CP010087–CP010088	E	BANN4	5,312,686	1	35
HD 1011	CP009332–CP009336	E	BANN3	6,093,375	4	35
HD 571	CP009599–CP009600	E	BANN8	5,312,179	1	35
HD 682	CP009717–CP009720	E	BANN8	5,291,389	3	35
subsp. Kurstaki HD 1	CP009998–CP010012	E	BANN16	6,859,374	14	35
subsp. *Morrisoni* HD 600^b^	JTHH00000000	E	BANN17	6,916,808	7	35
subsp. *thuringiensis* HD 1002	CP009344–CP009351	E	BANN15	6,572,702	7	35

^a^ If a strain is listed in the inclusivity panel, it is notated with an “I”; if it is in the exclusivity panel, it is notated with an “E.”

^b^ Strain *B. thuringiensis* subsp. *Morrisoni* HD600 is at Improved High Quality Draft (IHQD) status in 8 contigs, while all other genomes are completed to finished status (11).

### Nucleotide sequence accession numbers.

Accession numbers for all 35 genomes are listed in Table 1.
